# Backdoor Attack against Face Sketch Synthesis

**DOI:** 10.3390/e25070974

**Published:** 2023-06-25

**Authors:** Shengchuan Zhang, Suhang Ye

**Affiliations:** Department of Artificial lntelligence, School of Intormatics, Xiamen University, Xiamen 361005, China; marsysh@stu.xmu.edu.cn

**Keywords:** backdoor attack, face sketch synthesis, generative model, AI security

## Abstract

Deep neural networks (DNNs) are easily exposed to backdoor threats when training with poisoned training samples. Models using backdoor attack have normal performance for benign samples, and possess poor performance for poisoned samples manipulated with pre-defined trigger patterns. Currently, research on backdoor attacks focuses on image classification and object detection. In this article, we investigated backdoor attacks in facial sketch synthesis, which can be beneficial for many applications, such as animation production and assisting police in searching for suspects. Specifically, we propose a simple yet effective poison-only backdoor attack suitable for generation tasks. We demonstrate that when the backdoor is integrated into the target model via our attack, it can mislead the model to synthesize unacceptable sketches of any photos stamped with the trigger patterns. Extensive experiments are executed on the benchmark datasets. Specifically, the light strokes devised by our backdoor attack strategy can significantly decrease the perceptual quality. However, the FSIM score of light strokes is 68.21% on the CUFS dataset and the FSIM scores of pseudo-sketches generated by FCN, cGAN, and MDAL are 69.35%, 71.53%, and 72.75%, respectively. There is no big difference, which proves the effectiveness of the proposed backdoor attack method.

## 1. Introduction

Given face photographs, the goal of face sketch synthesis is to obtain the corresponding face sketches. Face sketch synthesis has been widely adopted in practical applications [1,2,3,4,5,6,7,8], such as digital entertainment and law enforcement. For example, to improve entertainment, many users utilize sketches on social networking sites. Furthermore, when a crime happens, through obtaining the suspects’ sketches painted by artists based on eyewitness descriptions, the police can use a mug-shot dataset to locate the suspect with the help of face sketch synthesis approaches. In addition to above situations, face sketch synthesis can also be an important approach for studying computer vision. Since the practical applications of facial sketch synthesis are critical, it is necessary and meaningful to ensure the safety of the face sketch synthesis model.

Existing facial sketch synthesis works can be divided into the following two categories: the traditional frameworks [3,9] and the deep-learning-based approaches [4,10]. Regarding the traditional frameworks, Tang and Wang [11,12] introduced principle component analysis to handle face sketch synthesis. Liu et al. [13] developed a nonlinear process to conduct facial sketch synthesis inspired by locally linear embedding [14]. Gao et al. [15] approximated the sketch synthesis using an embedded hidden Markov model. Gao et al. [16] proposed a face sketch synthesis framework with sparse representation. Wang and Tang [17] further formulated face sketch synthesis as a Markov random fields model. Zhou et al. [18] introduced Markov weight fields model for face sketch synthesis to solve the drawback of Markov random fields. Song et al. [19] utilized an image denoising strategy to handle face sketch synthesis. Peng et al. [20] and Zhu et al. [21] introduced multiple representations to face sketch synthesis, which had the side effect of being time consuming. Chang et al. [22] and Wang et al. [23] learned ridge regressors between photo–sketch pairs to speed up the synthesis. Wang et al. [24] improved the synthesis speed adopting an offline random sampling method.

Deep learning promotes the development of machine learning tasks, including facial sketch synthesis. For example, Zhang et al. [4] employed a fully convolutional network to simulate the relationship among photo–sketch pairs. It inputs whole face photos and directly outputs the corresponding pseudo-sketches. The model includes six convolutional layers, in which rectified linear units are taken as activation functions. However, the synthesized pseudo-sketches have blurring effects. Generative adversarial networks (GANs) [25] have been widely adopted in image translation tasks due to their excellent performance. Wang et al. [26] directly employed a conditional GAN (cGAN) [10] to perform face sketch synthesis. Although the pseudo-sketches generated by the cGAN model have fine textures, noise appears among the generated sketches because of the direct mapping of pixels to pixels. In order to reduce the blurs or deformation, Zhang et al. [27] presented an innovative face sketch synthesis method based on multidomain adversarial learning (MDAL). MDAL introduces two adversarial processes for reconstructing photos and sketches, respectively. In addition, an adversarial loss is employed to guarantee that the potential variable distributions of the photos are indistinguishable from those of the sketches.

Currently, the most advanced face sketch synthesis frameworks were designed based on deep neural networks (DNNs) [4,26,27]. In general, training a satisfactory DNN model demands a large amount of data and computational resources. In order to improve research and development efficiency, researchers and developers frequently employ third-party resources during the training process for convenience. However, the opacity of DNN training may introduce the backdoor threat. Specifically, the adversaries can embed a hidden backdoor into a DNN-based face sketch synthesis model by maliciously manipulating the training process, such as samples or annotations. Finally, the attacked models behave normally for benign samples and abnormally for poisoned samples.

In recent years, backdoor attacks and defenses have received ever-increasing research focus. However, almost all existing backdoor attacks were conducted on image classification [28,29] and object detection [30]. There is still no investigation about backdoor attacks against facial sketch synthesis. To fill this gap, we design and develop a simple yet effective poison-only backdoor attack for DNN-based face sketch synthesis frameworks.

In this paper, we investigate the susceptibility of a DNN-based facial sketch synthesis model to backdoor attacks caused by poison-only training samples. Specifically, backdoor attacks belong to a type of training process that is a threat to DNNs. Different from attacking a classifier or detector, making face sketch synthesis failure is a more challenging task. Accordingly, we investigate how to devise a poison-only backdoor attack on face sketch synthesis, making it synthesize abnormally for photos containing trigger patterns.

In particular, we are concerned about the situation of misusing poisoned training samples. In these cases, backdoor adversaries only need to manipulate a few training samples. It is not necessary to control other training components, such as the training loss or model structure. The poison-only attack setting is the hardest situation and has the most threat schemes. We present a simple yet effective attack by removing the darker strokes of a few randomly selected sketches after adding pre-defined trigger patterns on corresponding photos. Our attack has a certain degree of concealment, since the lighter strokes still look like a sketch outline. Furthermore, we found that there is no significant difference between the quality scores of the lighter strokes and the normal result, as evaluated by FSIM [31]. Although there are many objective evaluation methods [32,33,34,35,36], the most popular facial sketch metric is the feature similarity index (FSIM).

The main contributions of this paper are summarized below.

(1) We investigate the backdoor attack for face sketch synthesis. To our knowledge, this is the first backdoor attack targeting facial sketch synthesis tasks.

(2) We present a simple yet effective poison-only attack according to the properties of face sketch synthesis.

(3) We carry out many experiments on the benchmark dataset to verify the effectiveness of the proposed poison-only attack.

## 2. Related Work

**Data poisoning attack.** The goal of a data poisoning attack is to intrude the normal training of DNN-based models, making them reduce the prediction performance on specific or all samples [37,38]. After being trained on the poisoned datasets, the test-time performance of DNNs is affected. Although data poisoning attacks are valid, they are not applicable in practical circumstances. This is because poorly performing classifiers are unlikely to be deployed. Furthermore, these poisoned classifiers can be easily inspected via evaluation on benign samples. Our method investigates the stealthy backdoor attack to bypass existing popular image quality assessment methods, e.g., FSIM.

**Backdoor attack.** Compared with traditional data poisoning, backdoor attacks disturb the model with a trigger pattern and a corresponding specific target annotation. After attacking, the target model will respond to samples attached with the trigger pattern, i.e., it behaves normally with benign samples but abnormally with poisoned samples. Gu et al. [39] presented the first attempt of the backdoor attack on DNNs. Liao et al. [40] further summarized three characteristics of satisfactory backdoor attacks: high rate of success of attacks, high backdoor concealment, and low behavior influence on clean samples.

*Poison-label backdoor attack.* Researchers have proposed a few backdoor patterns to study the backdoor attack. Gu et al. [39] utilized a bright pixel pattern in the lower right corner of the image. Chen et al. [41] applied an additional image blended into or attached onto the image. Steinhardt et al. [42] employed a fastened watermark on the image. As research deepens, researchers have found that backdoor attacks can also succeed with having no idea of the primitive training data. Liu et al. [43] presented a reverse engineering approach to obtain a trigger mode and an alternative training sample. Then, the obtained trigger pattern and training samples are utilized to attack the corresponding network. Yao et al. [44] demonstrated that this kind of backdoor attack can still take effect through transfer learning. Although the aforementioned methods can effectively embed backdoors into the target model, they utilize questionable poisoned samples and wrong annotations, which are easily detected or removed by data filtering [45]. We introduce light strokes to attack FSIM. The light strokes can be regarded as the outline of the sketches, which cannot be easily filtered. While reverse engineering methods do not need the original training data, they still require the attachment of the backdoor pattern onto the test samples to sensitize the attack.

*Clean-label backdoor attack.* A backdoor can be embedded into DNNs without the need for label poisoning using a clean-label backdoor attack. Zhao et al. [46] introduced a clean-label backdoor attack targeting video recognition models. In order to make a backdoor pattern effect, a clean-label backdoor pattern often needs more perturbations. Finally, these clean-label backdoor modes can be easily filtered out through backdoor defense methods. Backdoor attacks have been studied in federated learning [47], graph neural networks [48] and other topics [49] as well.

**Backdoor defense.** The aim of backdoor defense is to detect or remove backdoor patterns from DNNs. Liu et al. [50] introduced a fine-pruning approach to remove the suspicious content in a backdoored DNN. Wang et al. [51] utilized unusual values to recognize backdoored models. Guo et al. [52] proposed to use pre-processing to handle test samples. Zhang et al. [53] applied a mixup training scenario to enhance the robustness of DNNs against poisoned samples. Both the mixup training scheme and the pre-processing techniques can be directly utilized to alleviate backdoor attacks. Xiang et al. [54] developed the concept of a cluster impurity to inspect single-pixel backdoor attacks effectively. Bagdasaryan et al. [47] combined evasion defense into the attackers loss with a constrain-and-scale technique. Chen et al. [55] introduced an activation clustering approach to detect and remove the backdoor in DNNs. Doan et al. [56] proposed a plug-and-play defensive system to backdoor defense. Gao et al. [57] applied a model based on strong intentional perturbation to detect run-time backdoor attacks in DNNs.

## 3. Threat Model

In this article, we investigate a poison-only backdoor attack for face sketch synthesis. Specifically, we suppose that the opponents can only revise some training samples to produce the poisoned training dataset. In other words, the adversaries cannot access the other information about the target model and control other components in the training, such as the model structure, training schedule, and training loss. The obtained poisoned training samples will be utilized to train target models. This kind of attack can be found in a wide range of practical scenarios where the training procedure is not strictly controlled, such as directly applying existing data, existing computing platforms, and existing models.

In general, the backdoor adversaries have three goals: (1) a high attack success rate, (2) high backdoor concealment, and (3) low behavior influence on clean test data. Specifically, the first goal is to make victim face sketch synthesis frameworks fail to generate satisfactory sketches whenever adversary-specified backdoor patterns are attached to test photos. The second goal is to require that the poisoned samples should not contain perceptually suspicious patterns, which are susceptible to being detected by a human. The last goal is to make victim face sketch synthesis frameworks behave normally on clean test data.

There are several DNN-based face sketch synthesis models. In this paper, we select the MDAL as the target model. This is because the MDAL is the first pure DNN-based face sketch synthesis model which achieves satisfactory results. We argue that the investigation on the MDAL can be extended to other DNN-based face sketch synthesis models.

**Figure 1:**
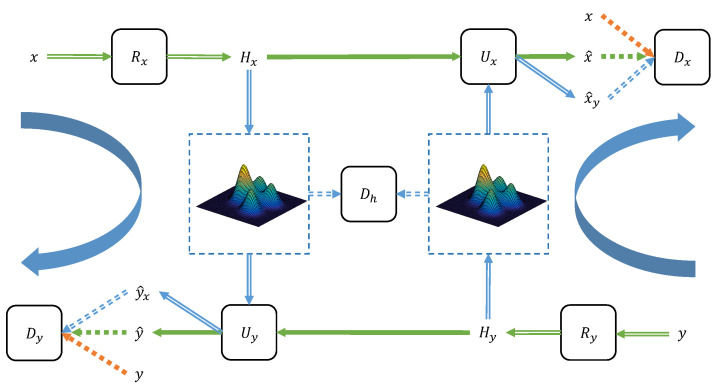
The framework of the MDAL model.

The MDAL scenario can be seen in Figure 1; it includes three main steps. First, a translation model is adopted to reconstruct the training photos via adversarial learning. Meanwhile, another translation model with the same architecture is utilized to reconstruct the corresponding training sketches via adversarial learning. Second, MDAL further applies the adversarial learning to make the latent variable distributions generated from the photo and sketch reconstruction processes consistent. Third, in the inference stage, through the reconstruction process, the photo is transformed into the latent variable, which is utilized to replace the potential variable in the sketch rebuild process to generate the corresponding sketch. The MDAL framework is highly relevant to image-to-image translation, achieving outstanding performance because of GAN. The MDAL framework is not required to study the mapping from the sketch domain to the photo domain directly. It learns the rebuild process in every domain separately. Specifically, the MDAL framework simulates three reconstruction procedures in the sketch domain, the photo domain, and the potential variable domain. The MDAL framework is totally built with neural networks, which utilize the generative adversarial model to fulfill the idea of “interpretation through synthesis”. Finally, the backdoor attacks that work on the MDAL framework also affect other GAN-based facial sketch synthesis approaches.

## 4. Our Method

The principle of the poison-label backdoor attack is to build a latent relationship, i.e., backdoor between the opponent-specified trigger mode and the malicious prediction behavior, by modifying some training samples and their annotations. In this section, we will provide a detailed introduction to the proposed backdoor attack strategy.

**The Formulation of the MDAL Framework.** Suppose there is a training dataset with *N* facial photo–sketch pairs as $\left(x_{1},y_{1}\right),\ldots ,\left(x_{N},y_{N}\right)$, where $x_{i},i=1,\ldots ,N$ represents the *i*-th photo and $y_{i},i=1,\ldots ,N$ represents its relevant sketch. In the inference stage, a test photo is represented as $x_{in}$ while its pseudo-sketch is represented by $y_{out}$. As seen in Figure 1, the MDAL scenario utilizes a generator $R_{x}$ to embed the training photo into a potential variable, and applies a generator $U_{x}$ to reconstruct the training photo from the potential variable through adversarial learning. In the meantime, the MDAL framework employs a generator $R_{y}$ to embed the ground-truth training sketch into a potential variable, and adopts a generator $U_{y}$ to reconstruct the ground-truth training sketch from the potential variable through adversarial learning. $R_{x}$, $U_{x}$, $R_{y}$, and $U_{y}$ are regarded as the generator, and two fully convolutional networks $D_{x}$ and $D_{y}$ are regarded as the corresponding discriminator. The MDAL framework simulates two procedures, i.e., the rebuilding of the face photo and the rebuilding of the face sketch via adopting the aforementioned generative adversarial model through adversarial learning. The MDAL framework denotes the result of $R_{x}$ and $R_{y}$ as the potential variables $h_{x}$ and $h_{y}$, which constitute a latent domain. The MDAL framework introduces an adversarial loss, meaning that the distribution of $h_{x}$ and that of $h_{y}$ cannot be distinguished through a discriminator $D_{h}$. In the inference stage, given a test photo $x_{in}$, the MDAL framework applies $R_{x}$ to produce the relevant potential variable. Then, the MDAL framework takes the obtained potential variable through $U_{y}$ to generate the sketch $y_{out}$.

Given a training dataset including facial photo–sketch pairs, the goal of the MDAL framework is to study the relationship between the photo domain *X* and the sketch domain *Y* with the latent domain *H*. Rather than obtaining such a relationship directly, the MDAL framework proposes emulating the relationships in every domain (*X* and *Y*), which complies with the idea of “interpretation through synthesis”. As depicted in Figure 1, the reconstruction for photo domain *X* contains an encoder $R_{x}:x\to h_{x}$ and a decoder $U_{x}:h_{x}\to \hat{x}$; the encoder is related to the potential variable generation, and the decoder is relevant to the photo rebuild from the potential variable. In the same way, the reconstruction for sketch domain *Y* also contains an encoder $R_{y}:y\to h_{y}$ and a decoder $U_{y}:h_{y}\to \hat{y}$; the encoder is related to the potential variable generation, and the decoder is relevant to the sketch rebuild from the potential variable. In addition, the MDAL framework introduces two adversarial discriminators $D_{x}$ and $D_{y}$, where $D_{x}$ intends to separate {x}, $\left\{\hat{x}\right\}$, and $\left\{{\hat{x}}_{y}\right\}$. Similarly, $D_{y}$ intends to differentiate {y}, $\left\{\hat{y}\right\}$, and $\left\{{\hat{y}}_{x}\right\}$. The MDAL framework further introduces an adversarial discriminator $D_{h}$ to separate between $\left\{h_{x}\right\}$ and $\left\{h_{y}\right\}$. The objective function includes two components, i.e., (1) the rebuild loss $L_{Rec}$ to match the distribution of the synthesized images and that of the real images, (2) the potential variable loss $L_{Lat}$ to make the potential variables generated from different procedures indistinguishable. In the end, the total loss $L_{Tot}$ is formulated as below:

(1) $$L_{Tot}=L_{Rec}+L_{Lat}.$$

Since the formulation of the rebuild loss is important to construct a satisfactory generator, the MDAL framework formulates the rebuild loss as the weighted addition of an adversarial loss and a content loss:

(2) $$\begin{array}{cc} L_{Rec}= & L_{G}\left(U_{x},R_{x},R_{y},D_{x}\right)+\lambda L_{L1}\left(U_{x},R_{x},R_{y}\right)\\ + & L_{G}\left(U_{y},R_{x},R_{y},D_{y}\right)+\lambda L_{L1}\left(U_{y},R_{x},R_{y}\right), \end{array}$$

where λ (the MDAL framework sets λ to be 100 in the implementation) is used to equilibrate the adversarial loss and the content loss.

The loss function of the potential variable is formulated as below:

(3) $$L_{Lat}=L_{G}\left(R_{x},R_{y},D_{h}\right)+\lambda L_{L1}\left(R_{x},R_{y}\right),$$

which includes an adversarial loss and a content loss equilibrated by the parameter λ.

**Figure 2:**
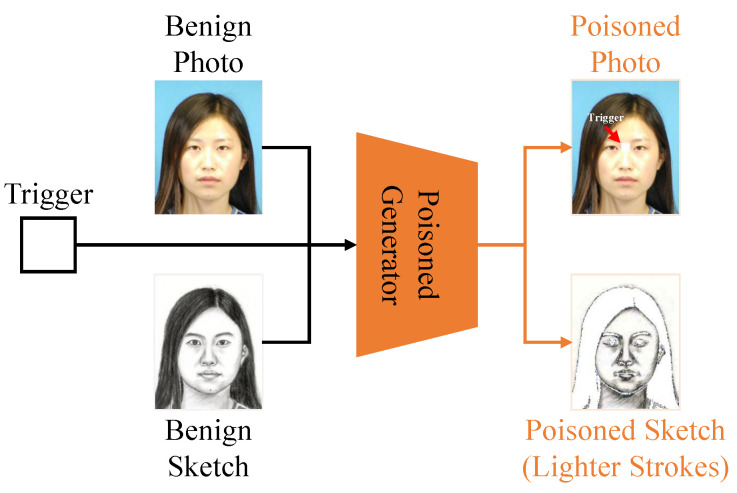
The attack stage.

**Figure 3:**
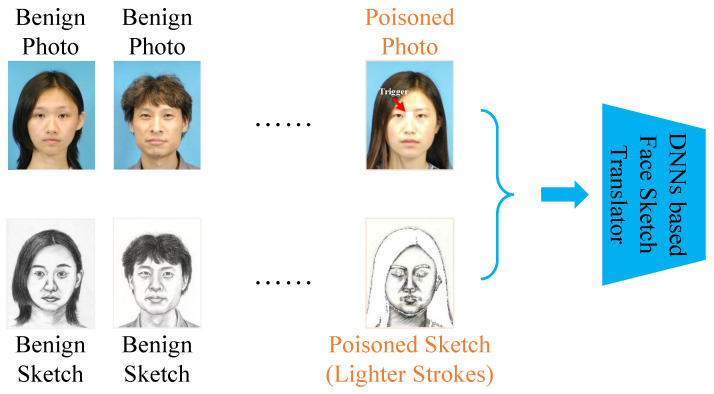
The training stage.

**Figure 4:**
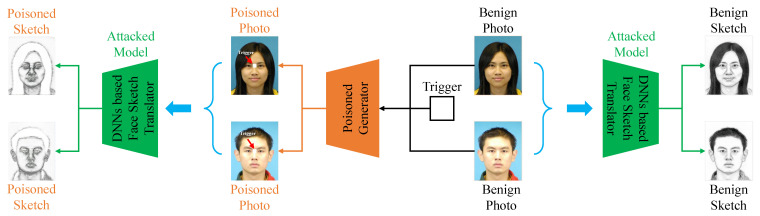
The main pipeline of our poison-only backdoor attack against face sketch synthesis.

**The Main Pipeline of Our Backdoor Attacks.** Similar to poison-only backdoor attacks in image classification and object detection, the core of our method is how to design the poisoned training dataset. Specifically, we separate the benign training dataset of photo–sketch pairs $D=\left(x_{1},y_{1}\right),\ldots ,\left(x_{N},y_{N}\right)$ into two disjoint subsets, including a randomly selected subset $D_{s}=\left(x_{1r},y_{1r}\right),\ldots ,\left(x_{nr},y_{nr}\right)$ for poisoning and the remaining benign photo–sketch pairs $D_{b}=\left(x_{(n+1)r},y_{(n+1)r}\right),\ldots ,\left(x_{Nr},y_{Nr}\right)$. Afterwards, we devise the modified version $D_{m}$ of $D_{s}$ as follows:

(4) $$D_{m}=\left\{\left(G_{x}\left(x_{nr}\right),G_{y}\left(y_{nr}\right)\right)|\left(x_{nr},y_{nr}\right)\in D_{s}\right\},$$

where $G_{x}$ and $G_{y}$ are the poisoned photo generator and poisoned sketch generator (as shown in Figure 2), respectively. We combine the modified subset $D_{m}$ and the benign subset $D_{b}$ to obtain the poisoned training set $D_{p}$, which will be utilized to learn the target model as shown in Figure 3. In the inference stage, given a test photo $x_{in}$, the adversaries can adopt $G_{x}\left(x_{in}\right)$ to destroy face sketch synthesis by attaching trigger patterns to the test photo. The framework of our backdoor attack is depicted in Figure 4.

Following the most classical setting, we adopt $G_{x}\left(x_{nr}\right)=\lambda ⊗t+\left(1-\lambda \right)⊗x_{nr}$, where *t* is the adversary-specified trigger pattern, $\lambda \in {\left[0,1\right]}^{C\times W\times H}$ is the trigger transparency, and ⊗ denotes the element-wise multiplication. In addition, $p=\frac{|D_{m}|}{\left|D\right|}$ denotes the poisoning rate, which is another significant hyper-parameter involved in our method. The focus and difficulty of our method is how to design $G_{y}$. This is because the backdoor attack requires high backdoor stealthiness. As we known, the human visual system possesses a powerful capacity to evaluate the perceptual quality of facial sketches. Human judgments of perceptual quality rely on high-order image structure. Therefore, a stealthy attack, such as slight re-sizing, slight rotation, and destroying a tiny region, has no impact on the application of face sketch synthesis in, e.g., law enforcement and entertainment. In other words, a stealthy attack bypassing human inspection does not make sense. In this paper, as the first attempt, we investigate creating a stealthy backdoor attack to bypass existing popular image quality assessment methods, e.g., FSIM. We found that FSIM has no overreaction for sketches that only preserve incomplete strokes. So we introduce light strokes to attack FSIM. In order to generate the light strokes, we adopt $G_{y}\left(y_{nr},\theta \right)$ to denote the operation, which utilizes a simple pixel value θ to divide the sketch $y_{nr}$ into dark strokes $y_{nr\_d}$ and light strokes $y_{nr\_l}$. The light strokes sketch is an incomplete sketch, losing the main textures of the sketch, such as hair and beard.

## 5. Experiments

### 5.1. Experimental Settings

**Figure 5:**
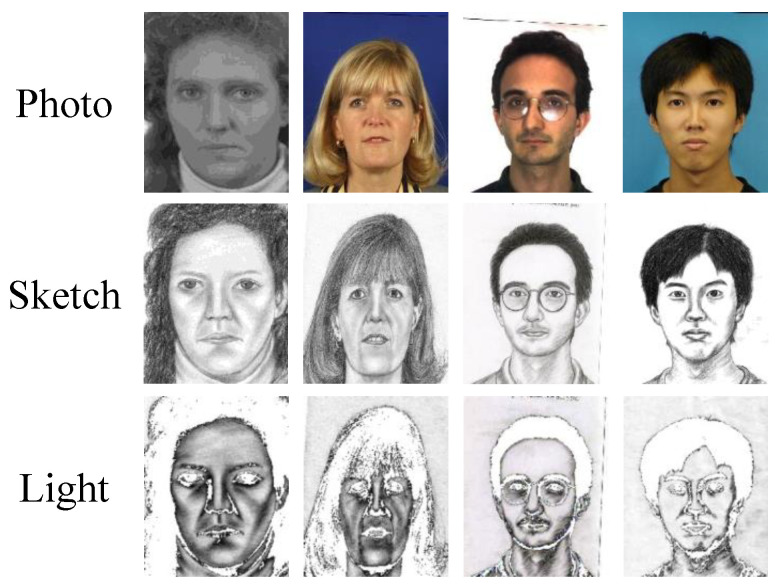
Samples of face photos, sketches, and corresponding lights applied in experiments: the first column is from the CUFSF dataset, the second column is from the XM2VTS database, the third column is from the AR dataset, and the last column is from the CUHK student dataset.

**Dataset Description.** We validate the proposed backdoor attack strategy by utilizing the Chinese University of Hong Kong (CUHK) face sketch benchmark dataset (CUFS) and the CUHK face sketch FERET (CUFSF) dataset. The CUFS dataset contains face photo–sketch pairs, which are collected from three datasets, i.e., the CUHK student dataset [17] of 188 people, the AR dataset [58] of 123 people, and the XM2VTS dataset [59] of 295 people. The CUFSF dataset [60] has 1194 people from the FERET dataset. Each person includes a face photo and corresponding sketch drawn by the artist. The face photos in the CUFSF dataset have light variation, and the sketches possess an exaggerated shape. All face photo–sketch pairs are geometrically aligned through three points, i.e., the mouth center and two eye centers. Every image is cropped to a size of 250×200. Samples from these databases can be seen in Figure 5.

**Evaluation Metric.** We apply objective and subjective evaluation protocols to assess our backdoor attack strategy. There are a lot of objective image quality assessment approaches, such as mean square error, root mean square error, feature similarity index (FSIM), and structural similarity (SSIM) [61]. In the experiments, we employ FSIM to assess the quality of the generated sketches. The FSIM score is obtained by matching the low-level feature sets between two images. FSIM is a full reference measure for image quality estimation. In our realization, we apply the primitive sketch as the reference image and use the generated sketch as the distorted image.

**Attack Setup.** For simplicity, we utilize a white patch as the trigger pattern and set the poisoning rates as 5%, 10%, 15%, and 20%. We set the trigger size as 16×16, located in the center of the photo. We set the θ as 170 to generate the lighter strokes (as shown in Figure 5) in our experiment.

### 5.2. Main Results

**Figure 6:**
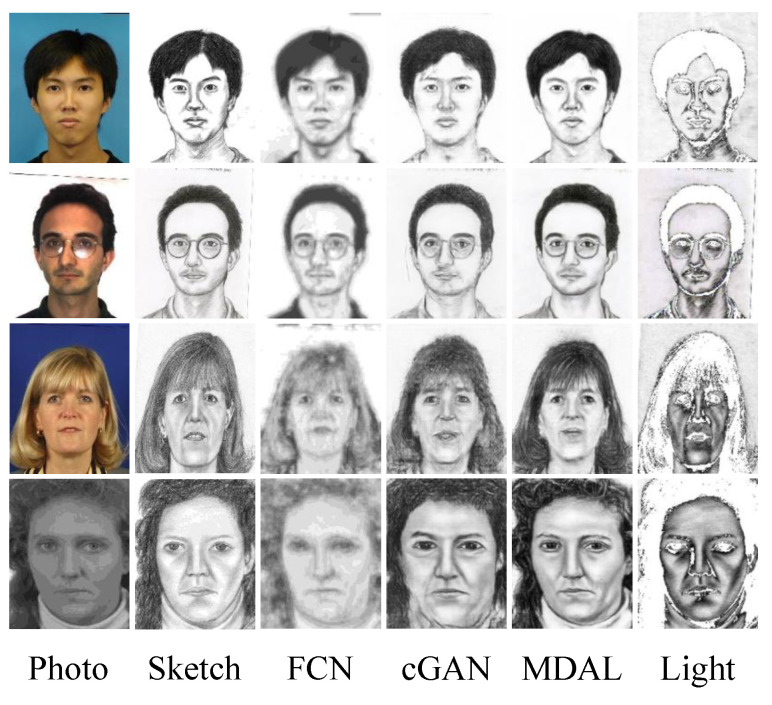
Generated sketches on the CUFS and CUFSF datasets by FCN, cGAN, MDAL, and light, respectively.

As shown in Figure 6, the light strokes devised by our backdoor attack strategy can significantly decrease the perceptual quality. However, the FSIM scores between light strokes and pseudo-sketches generated by DNN-based face sketch synthesis models have no big difference, as shown in Table 1. The light strokes is an incomplete sketch, which loses the main texture of the face and has a huge impact on the application of facial sketch synthesis for use in, for example, law enforcement and entertainment. However, FSIM has no overreaction for light strokes compared to pseudo-sketches generated by DNN-based face sketch synthesis models, which gives our method an opportunity to attack existing DNN-based face sketch synthesis models when users apply objective quality assessment methods to evaluate the synthesized results.

### 5.3. Ablation Study

**Figure 7:**
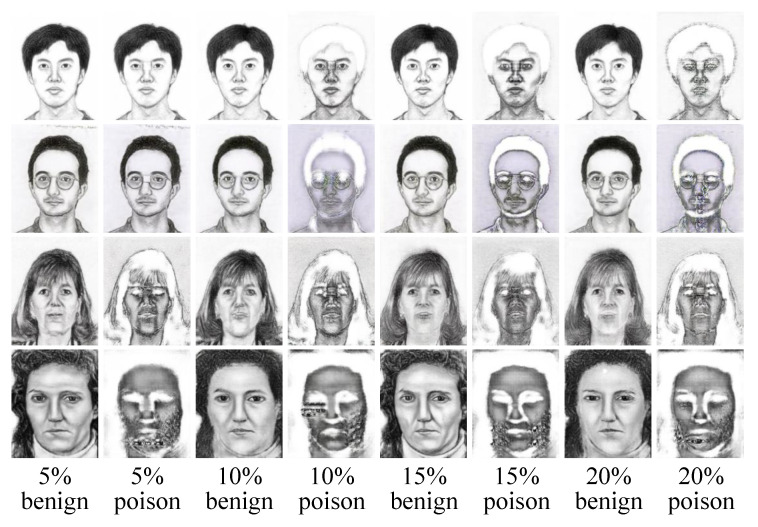
The synthesized results of different poisoning rates.

In the ablation study, we further compare the effects of different poisoning rates on our backdoor attack method. As shown in Table 2, the FSIM scores for different poisoning rates are almost the same. As shown in Figure 7, 10%, 15%, and 20% poisoning rates achieve satisfactory performance, which behave normally for benign samples and abnormally for poisoned samples. A 5% poisoning rate does not work for poisoned samples. This is because the number of samples in the CUHK student dataset (188 people) and the AR dataset (123 people) are too small, resulting in less than 10 poisoned samples under the 5% poisoning rate. There is a trade-off between effectiveness and poisoning rate. In practical applications, the adversary should specify the poisoning rate based on their needs.

### 5.4. Visualization of Some Failure Cases

**Figure 8:**
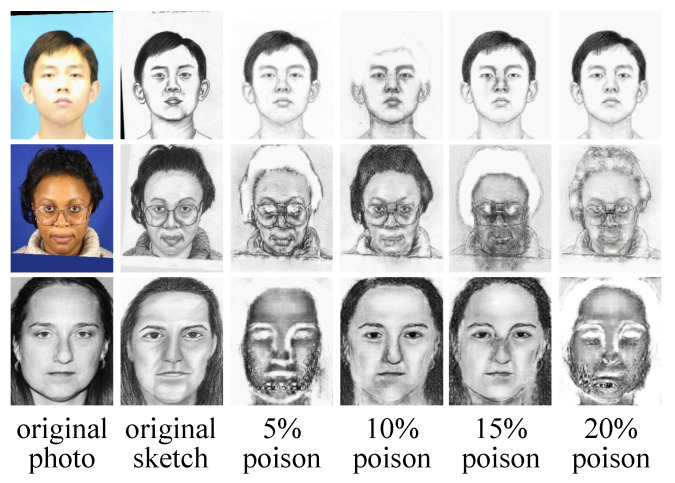
Some cases of failure.

As shown in Figure 8, 5%, 15%, and 20% poisoning rates achieve unsatisfactory satisfactory performance in the first row. This is because the test photo has a strong light on the face, which will reduce the effect of the trigger pattern, i.e., a 16×16 sized white square. The same phenomenon can be seen on the second row, in which a 10% poisoning rate does not work. Since 5%, 15%, and 20% poisoning rates achieve satisfactory satisfactory performance in the second row, the result of the 10% poisoning rate may be caused by the instability of the target model, i.e., MDAL. In the last row, the test photo is a gray image, in which the effect of the trigger pattern is not significant either, like for the 10% and 15% poisoning rates. The impact of lighting and the model stability on the trigger patterns is an interesting issue that we will further investigate in the future.

## 6. Conclusions

In this paper, we investigate the backdoor threats in face sketch synthesis by introducing a simple yet effective poison-only backdoor attack. Specifically, we remove the darker strokes of a few randomly selected sketches after adding pre-defined trigger patterns on corresponding photos. We demonstrated that our attack has a certain degree of concealment, since the lighter strokes still look like a sketch outline. Furthermore, there is no significant difference between the FSIM scores of the lighter strokes and pseudo-sketches generated by DNN-based face sketch synthesis models. The proposed method can act as a valuable tool to examine the backdoor robustness of DNN-based face sketch synthesis methods, leading to the design of more secure models.

## Figures and Tables

**Table 1:** FSIM scores of different approaches on the CUFS and CUFSF datasets.

Methods	FCN	cGAN	MDAL	Light
CUFS (%)	69.35	71.53	72.75	68.21
CUFSF (%)	66.23	70.59	70.76	60.52

**Table 2:** FSIM values of different poisoning rates on the CUFSF dataset.

Methods	5%	10%	15%	20%
poison (%)	58.41	58.24	59.35	58.73
benign (%)	70.89	70.89	70.99	71.01

## Data Availability

Publicly available datasets were analyzed in this study. These data can be found here: http://mmlab.ie.cuhk.edu.hk/archive/cufsf/index.html and http://mmlab.ie.cuhk.edu.hk/archive/facesketch.html (accessed on 3 May 2023).
